# Classification of anatomy and treatment approaches for aneurysms originating from the proximal of the A1 segment of the anterior cerebral artery in clinical settings

**DOI:** 10.3389/fneur.2024.1369414

**Published:** 2024-07-23

**Authors:** Xiao-meng Liu, Xiao-lei Song, Kai Tang, Chao Zhang, Xiao-song Liu, Lei Zhao, Xiao-liang Wang, Hai-long Du, Yu-hua Hu, Jian-liang Wu

**Affiliations:** Department of Neurosurgery, The Second Hospital of Hebei Medical University, Shijiazhuang, China

**Keywords:** aneurysms originating from the A1 segment, anterior cerebral artery, clinical types, endovascular treatment, microsurgical clipping

## Abstract

**Objective:**

To explore the spatial relationship between A1 segment proximal anterior cerebral artery aneurysms and their main trunks, classify them anatomically and develop targeted treatment strategies.

**Methods:**

This single-center retrospective analysis involved 39 patients diagnosed with aneurysms originating from the proximal of A1 segment of the anterior cerebral artery (2014–2023). Classify the patient’s aneurysm into 5 types based on the location of the neck involving the carrier artery and the spatial relationship and projection direction of the aneurysm body with the carrier artery, and outcomes from treatment methods were compared.

**Results:**

Among 39 aneurysms, 18 cases underwent endovascular intervention treatment, including 6 cases of stent assisted embolization, 1 case of flow-diverter embolization, 5 cases of balloon assisted embolization, and 6 cases of simple coiling. At discharged, the mRS score of all endovascularly treated patients was 0, and the GOS score was 5 at 6 months after discharge. At discharge, the mRS score of microsurgical clipping treated patients was 0 for 15 cases, 3 for 1 case, 4 for 1 case and 5 for 2 cases. Six months after discharge, the GOS score was 5 for 16 cases, 4 for 2 cases, 3 for 2 cases, and 1 for 1 case. GOS outcomes at 6 months were better for endovascularly treated patients (*p* = 0.047).

**Conclusion:**

Results showed better outcomes for the endovascular treatment group compared to microsurgical clipping at 6 months after surgery. The anatomical classification of aneurysms in this region may be of help to develop effective treatment strategies.

## 1 Introduction

Aneurysms located at the internal carotid bifurcation are denoted as aneurysms of the segment (A1) of the anterior cerebral artery. These intracranial aneurysms occurring in the region between the internal carotid arterial bifurcation (ICA) and anterior communicating artery (AcoA), constitute a rare subset, comprising merely 0.59–4% of all intracranial aneurysms. Notably, aneurysms originating from the A1 segment of the anterior cerebral artery exhibit an increased proclivity for rupture even at smaller diameters when compared to aneurysms in other cerebral regions. This propensity results in significant subarachnoid hemorrhage (SAH) (1), secondary severe cerebral vasospasm, epilepsy, brain tissue edema (2), and a strong correlation with the perforating artery. Furthermore, A1 segment aneurysms are frequently concomitant with multiple aneurysms in diverse cerebral regions and anatomical aberrations within intracranial vessels (3). As a result, treating A1 segment aneurysms poses a notable challenge for neurosurgeons.

Classification of A1 segment aneurysms has been approached diversely in existing literature and most A1 aneurysms exhibit directional preferences (4). Yasargil (5) initially classified these aneurysms into three types based on the proximal, middle, and distal thirds of the A1 segment. Subsequently, Ding et al. (6) expanded upon this classification, delineating six subtypes considering factors such as the basal position of the aneurysm, direction of the aneurysm body, and its relationship with perforator vessels. The identified subtypes include IA (posterior-inferior proximal), IB (posterior-superior proximal), IC (anterior-proximal), IIA (distally located at the bifurcation of the A1 segment with non-normal cortical branch or fenestration malformation), IIB (distal trunk of the A1 segment), and III (fusiform or dissecting aneurysm located at any part of the A1 segment), each requiring distinct treatment recommendations. Nevertheless, challenges emerge during interventions due to the proximate location of the initial segment of the A1 to the bifurcation of the internal carotid artery. This proximity complicates the attainment of super selection with a microcatheter and the subsequent maintenance of its stability post-selection. Consequently, these complexities elevate the risks associated with intraoperative rupture and bleeding. Additionally, microsurgical clipping procedures for A1 segment-originating aneurysms are hindered by their frequent posterior orientation (4, 7), making the isolation of their necks along with surrounding perforating vessels a challenging endeavor. Consequently, we focused exclusively on the anatomical types and treatment concepts pertaining to aneurysms originating from the proximal A1 segment. A retrospective analysis was conducted on 39 patients with proximal A1 segment aneurysms admitted to the Department of Neurosurgery at the Second Hospital of Hebei Medical University between May 2014 and June 2023. We collected, analyzed, and compared imaging data, clinical data, clinical prognosis, and follow-up data of the patients. Furthermore, a anatomical classification was conducted, leading to the formulation of distinct treatment approaches tailored to various clinical categories of proximal A1 segment-originating aneurysms.

## 2 Materials and methods

### 2.1 General information

The study cohort included 39 patients with aneurysms originating from the proximal A1 segment of the anterior cerebral artery, who were admitted to the Department of Neurosurgery at the Second Hospital of Hebei Medical University from May 2014 to June 2023. All enrolled patients underwent diagnostic assessments, including digital subtraction angiography (DSA), CT angiography (CTA), and/or magnetic resonance angiography (MRA), to confirm the presence of aneurysms originating from the proximal A1 segment.

### 2.2 Imaging examination

All patients received a diagnosis of intracranial aneurysms through 3D-CTA or DSA. Cases involving intracranial aneurysms attributed to alternate etiologies, such as intracranial vascular malformations, trauma, or moyamoya disease, were systematically excluded. Subsequently, three-dimensional images were reconstructed from the processed 3D-CTA/DSA data to delineate the anatomical types of aneurysms originating from the proximal A1 segment.

### 2.3 Anatomical subtype classification of proximal A1 segment aneurysm

The aneurysms originating from the beginning of A1 segment were systematically classified into five distinct types. Type I denotes an aneurysm with its neck positioned in the posterior wall of the parent artery, and the aneurysm body projecting rearward. Type I further subdivides into two subtypes: Type Ia, characterized by a backward and inferior projection of the aneurysm body, and Type Ib, distinguished by a superior posterior projection. Type II features an aneurysm with its neck located on the upper wall of the parent artery, and its body projecting upward. Type III is identified by an anterior location of both the aneurysm neck and the forward projection of its body along the anterior wall of the parent artery. Type IV is characterized by a lower neck position within the inferior wall of the parent artery, accompanied by a downward projection of its body. Lastly, Type V encompasses fusiform or large-sized aneurysms that involve more than two quadrants in cross-section or have a diameter exceeding 25 mm, with concurrent involvement at their necks.

### 2.4 Microsurgical clipping and intraoperative evaluation

The microsurgical methodologies implemented in this study adhered to the standard pterional approach, incorporating tailored modifications or expansions as warranted by individual patient conditions. A consistent practice across all cases involved employing an ipsilateral approach. After completing the routine craniotomy process under general anesthesia, we opened the lateral fissure and carotid artery pool, found the bifurcation of the end of the internal carotid artery and the origin of the anterior cerebral artery, dissected the M1 segment of the middle cerebral artery and the proximal part of the A1 segment of the anterior cerebral artery, followed the shape of A1 to search for the aneurysm, determined the base and direction of the aneurysm, and combined with preoperative imaging results, anatomically classified the aneurysm which located proximal A1 segment. Before clipping the aneurysm, dissect the distal A1 portion of the aneurysm to correctly identify and dissect the Heubner recurrent artery and perforating vessels originating from the A1 segment, in order to safely use temporary occlusion clips if necessary. The aneurysm clips used for clipping surgery mainly come from Sugita Aneurysm Clip and Yasargil. Furthermore, intraoperative indocyanine green angiography (ICGA) was used to assess the clipping of the aneurysm neck and the preservation of perforating vessels.

### 2.5 Endovascular interventional therapy and intraoperative evaluation

All patients underwent intravascular intervention treatment under general anesthesia. The patient inserted a 6F guide sheath through the femoral artery, and after systemic heparinization, a 6F guide tube was placed in the internal carotid artery (ICA) to the vertical petrous segment of the internal carotid artery. Throughout the entire surgical process, the activated coagulation time remained 2–3 times the baseline. We often use 0. 017 inch diameter microcatheters [Headway (Microinvention, Tustin, CA, United States) and low profile visualized intraluminal support (LVIS) stents (Microinvention, Tustin, CA, United States) and Tubridge] to embolize aneurysms and select stents based on the specific condition of the aneurysm (MicroPort^®^, Shanghai, China) and the manufacturer’s recommended stent delivery microcatheter are used to transport the stent. Firstly, perform 3D reconstruction of blood vessels and select the appropriate angle as the working angle; then, based on the shape of the blood vessels and the direction of the aneurysm, the tip of the microcatheter is steam molded to facilitate the smooth entry of the microcatheter into the aneurysm cavity and provide strong support. At first, when the patients had a ruptured aneurysm, the risk of rebleeding due to postoperative antiplatelet and anticoagulant therapy was sometimes considered. Therefore, in some cases, we adopted balloon assisted embolization. Later, with the improvement of surgical techniques and confidence in the ability to tightly embolize aneurysms during surgery, we adopted more stent assisted embolization methods. For emergency patients who use stent assisted embolization technology during surgery, postoperative intravenous injection of tirofiban hydrochloride is routine. Firstly, administer a load dose via intraductal arterial administration 0.4 μg/(kg·min) for 30 min (total dose not exceeding 1 mg), followed by intravenous infusion of 0.1 μg/(kg·min) maintaining for 24–48 h, then switch to oral aspirin (100 mg/d) and clopidogrel (75 mg/d). Patients with unruptured aneurysms were given aspirin (100 mg/d) and clopidogrel (75 mg/d) orally for 5–7 days before surgery. Thromboelastography showed that the patient’s platelet inhibition rate met the standard before surgery. All patients who underwent stent assisted embolization underwent routine oral administration of aspirin (100 mg/d) and clopidogrel (75 mg/d) for 3 months after surgery. After DSA examination without complications such as vascular stenosis, they were switched to long-term oral administration of aspirin (100 mg/d).

### 2.6 Postoperative evaluation methods and neurological function follow-up

The Glasgow Outcome Score (GOS) was used as the metric for assessing neurological function in all patients at various time points: preoperatively, at discharge, and at 3 and 6 months postoperatively. In the GOS scores of each subtype, a score range of 4–5 indicates a good prognosis, while a score range of 1–3 indicates a poor prognosis. And compare and analyze the recovery of neurological function.

### 2.7 Statistical analysis

All the data collected in this study were analyzed using SPSS 23.0. Normally distributed measurement data were expressed as mean ± standard deviation (SD), while non-normally distributed measurement data were expressed as median (interquartile range), and the comparisons were examined by Student-*t* test and Mann–Whitney test (non-parametric distribution). The categorical data were expressed as *n*(%), and the differences between the two groups were examined by chi-square analysis or Fisher’s exact test. *p* < 0.05 was considered statistically significant.

## 3 Results

### 3.1 Age, gender, and other demographic profiles

From May 2014 to June 2023, the Department of Neurosurgery at the Second Hospital of Hebei Medical University diagnosed and treated a group comprising 39 patients with aneurysms originating from the proximal A1 segment of the anterior cerebral artery. Among these cases, endovascular intervention was implemented for 18 patients (46.1%), while microsurgical clipping was applied for 21 patients (53.8%). The age distribution of the patients ranged from 8 to 69 years, with a mean age of 56.5 ± 11.32 years. The gender distribution indicated 24 females (61.5%) and 15 males (38.4%). Of the diagnosed cases, 25 (64.1%) were attributed to SAH resulting from aneurysm rupture, with 21 cases (53.8%) specifically originating from the A1 segment. Additionally, 15 cases (38.5%) presented with multiple aneurysms. The patients with SAH exhibited varying Hunt-Hess grades: Grade I – 1 case (2.5%), Grade II – 19 cases (48.7%), Grade III – 4 cases (10.2%), and Grade IV – 1 case (2.5%). Regarding the anatomical location, left-sided aneurysms originating from the proximal A1 segment constituted 19 cases (48.7%), while right-sided ones accounted for 20 cases (51.2%). Further delineation by gender revealed 7/8 male left-sided/right-sided cases and 12/12 female left-sided/right-sided cases (Table 1).

**Table 1:** Characteristics of patients presenting A1 segment aneurysms.

Characteristics	No. of cases*
Total number of cases	39
Number of cases treated	39
Intervention/clipping	18(46.1)/21(53.8)
Average age (Y)	56.5 ± 11.32
Male/Female, *n* (%)	15(38.5)/24(61.5)
Multiple aneurysms, *n* (%)	15(38.5)
Hunt-Hess grade	
No subarachnoid hemorrhage	14(35.8)
Subarachnoid hemorrhage, *n* (%)	25(64.2)
I, *n* (%)	1(2.5)
II, *n* (%)	19(48.7)
III, *n* (%)	4(10.2)
IV, *n* (%)	1(2.5)
Aneurysm size	
≤5 mm, *n* (%)	32(82.0)
5-25 mm, *n* (%)	5(12.8)
>25 mm, *n* (%)	2(5.1)
Aneurysm morphology	
Fusiform aneurysm	1(2.6)
Giant aneurysm	2(5.1)
Aneurysm location	
Left-sided/right-sided, *n* (%)	19(48.7)/20(51.2)
Preoperative and postoperative imaging features	
Preoperative IVH, *n* (%)	8(29.8)
New postoperative ICH, *n* (%)	3(7.8)
New postoperative IVH, *n* (%)	2(5.1)
Postoperative intracranial infection, *n* (%)	1(2.6)
Postoperative new ischemic cerebral infarction, *n* (%)	6(15.4)
Postoperative hydrocephalus, *n* (%)	2(5.1)

*Data are presented as the mean ± SD, or number of patients (%).

### 3.2 Imaging features

Based on imaging and intraoperative observations, preoperative head CT scans identified intraventricular hemorrhage (IVH) in 8 cases (29.8%). Among these, 1 case (2.6%) presented with a fusiform aneurysm involving the distal 1/3 of the A1 segment of the anterior cerebral artery, and 2 cases (5.1%) exhibited giant aneurysms. The remaining aneurysms were of a saccular nature. Aneurysm sizes were distributed as follows: ≤5 mm in 32 cases (82.0%), 5–25 mm in 5 cases (12.8%), and a total of 2 cases with sizes exceeding 25 mm (5.1%) (Table 1). Ten cases (25.6%) manifested contralateral absence or hypoplasia of the A1 segment. Post-surgery, 3 cases (7.8%) experienced intracerebral hemorrhage, and 2 cases (5.1%) presented with IVH.

### 3.3 Postoperative complications

Following the surgical intervention, a postoperative intracranial infection was documented in one case (2.6%). Additionally, new ischemic cerebral infarctions were identified in six cases (15.4%), and postoperative hydrocephalus manifested in 2 cases (5.1%) (Table 1).

### 3.4 Anatomical types and surgical methods of aneurysms originating from the proximal A1 segment of anterior cerebral artery

**Figure 1:**
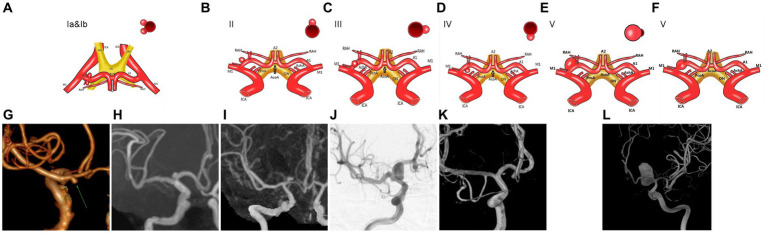
The aneurysm located at the beginning of the A1 segment of the anterior cerebral artery is classified clinically. **(A,G,H)** An illustration of type Ia and type Ib aneurysms of the A1 segment. **(B,I)** Illustration of A1 segment aneurysms of type II. **(C,J)** Illustration of A1 segment aneurysms of type III. **(D,K)** Illustration of A1 segment type IV aneurysms. **(E,F,L)** Illustration of A1 segment aneurysms of type V.

Following a thorough examination of the existing literature and a detailed analysis of the imaging and intraoperative data pertaining to the 39 patients presenting with aneurysms originating from the proximal A1 segment, the study cohort was systematically classified into five distinct groups based on the spatial relationship between the aneurysm neck and the parent artery. The rupture status, different surgical methods, postoperative complications, and prognosis of 5 different types of aneurysms were compared. The selection of treatment methods was influenced by various factors, such as the willingness of the patients’ family, the financial condition, and the surgeons’ experience, as detailed in Table 2 and illustrated in Figure 1.

**Table 2:** The prognosis after surgery among patients with various clinical types.

Surgical methods	Clinical types	Number of cases (*n*, %)	Good prognosis (*n*, %)	Poor prognosis (*n*, %)
Clipping	Ia	12 (30.8)	10 (25.6)	2 (5.1)
	Ib	5 (12.8)	5 (12.8)	0 (0)
	II	0 (0)	0 (0)	0 (0)
	III	2 (5.1)	1 (2.6)	1 (2.6)
	IV	1 (2.6)	1 (2.6)	0 (0)
	V	1 (2.6)	1 (2.6)	0 (0)
Endovascular treatment	Ia	6 (15.4)	6 (15.4)	0 (0)
	Ib	4 (10.3)	4 (10.3)	0 (0)
	II	6 (15.4)	6 (15.4)	0 (0)
	III	1 (2.6)	1 (2.6)	0 (0)
	IV	0 (0)	0 (0)	0 (0)
	V	1 (2.6)	1 (2.6)	0 (0)
Total no. of cases		39 (100)	36 (92.3)	3 (7.7)

#### 3.4.1 Type I

**Figure 2:**
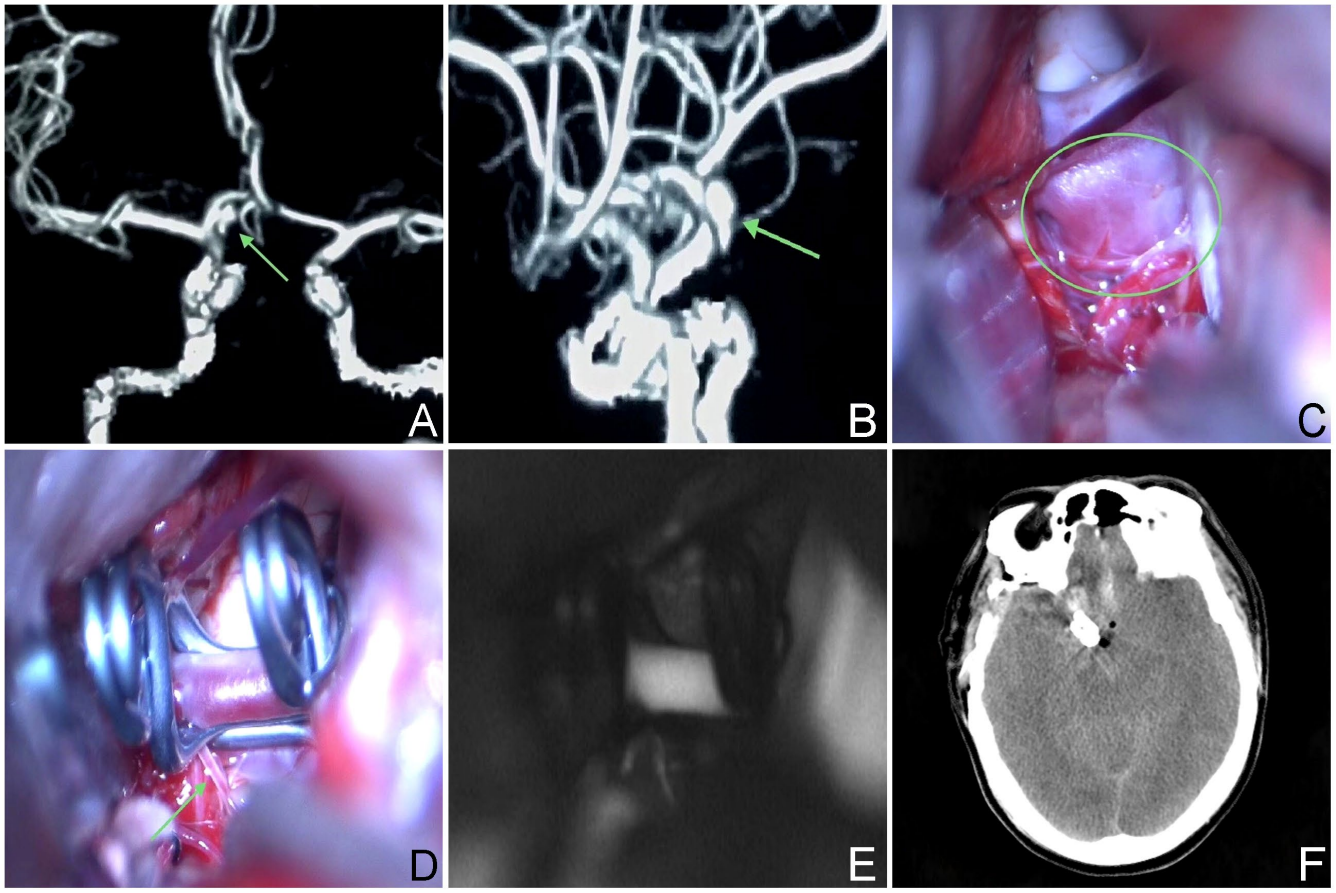
The aneurysm neck is situated within the posterior wall of the parent vessel. **(A,B)** A 59-year-old male, had an aneurysm that has not ruptured. The CTA revealed that the aneurysm was situated in the posterior wall of the parent artery. **(C–E)** Intraoperative situation. **(F)** Postoperative computed tomography scan.

The aneurysm neck was predominantly situated in the posterior wall of the parent artery, with 27 cases (59.1%) demonstrating a posterior projection of the aneurysm body, representing the most prevalent type within the identified categories. This classification yielded two distinct subtypes based on the direction of projection: Type Ia, characterized by a backward and inferior projection of the aneurysm in 18 cases (46.1%), and Type Ib, featuring a superior posterior tumor projection in 9 cases (23.0%) (refer to Figure 2).

Surgery was conducted on 27 patients (59.1%) with type I aneurysms, comprising 18 cases (46.1%) classified as Type Ia. Among these cases, 12 (30.8%) underwent microsurgical clipping. Notably, 10 patients (25.6%) exhibited a favorable recovery outcome, while 2 patients (5.1%) experienced poor recovery. Furthermore, 1 patient demonstrated moderate disability, and another patient exhibited severe disability upon discharge. Endovascular treatment was administered to 6 patients (15.4%), involving stent-assisted embolization in 3 cases, balloon-assisted embolization in 1 case, and thermoplastic single microtubule embolization in 2 cases. Immediate postoperative angiography revealed complete occlusion in all 6 treated patients, who subsequently experienced successful recovery and were discharged from the hospital. Nine cases (23.0%) belonged to Type Ib aneurysms; among them, 5 cases were managed through microsurgical clipping, while 4 cases underwent endovascular treatment, including single microcatheter embolization in 1 case, double microcatheter embolization in another case, and balloon-assisted embolization in 2 cases, respectively. All 4 treated patients exhibited complete occlusion based on immediate postoperative angiography findings. Overall, the prognosis for both surgical approaches was favorable. In the Type I microsurgical occlusion group, 5 cases experienced ipsilateral frontal lobe or basal ganglia cerebral infarction, with 1 case experiencing decreased muscle strength in one limb. After rehabilitation training, they were able to live independently and their muscle strength significantly improved. In the endovascular treatment group, 1 case experienced basal ganglia infarction but no neurological deficits was detected. A patient who underwent interventional treatment but was unable to successfully select a microcatheter and switched to microsurgical clipping experienced contralateral thalamic hemorrhage breaking into the ventricles for extracerebral drainage, followed by intracranial infection and hydrocephalus. After the bleeding and infection were cured, a lumbar cistern abdominal shunt was performed, and the patient suffered with moderate disability after surgery; 1 case presented with mild hydrocephalus and received conservative treatment (Table 3).

**Table 3:** The complications among patients with various clinical types.

Clinical types	Surgical methods	Ruptured (*n*)	Unruptured (*n*)	Complications
Ia	Clipping	10 cases (7 cases of grade II, 2 cases of grade III, and 1 case of grade IV)	2	4 cases of cerebral infarction; 3 case of asymptomatic cerebral infarction; 1 case of increased postoperative ventricular hematoma accompanied by hydrocephalus; 1 case of postoperative hydrocephalus complicated with intracranial infection
	Endovascular treatment	6 cases (1 case of grade I and 5 cases of grade II)	0	1 case of asymptomatic cerebral infarction
Ib	Clipping	Both cases are grade II	3	None
	Endovascular treatment	1 case of Grade II	3	None
II	Endovascular treatment	3 cases of grade II	3	None
III	Clipping	2 cases of grade III	0	Postoperative left frontal hematoma accompanied by pulmonary embolism and cardiac dysfunction
	Endovascular treatment	0	1	None
IV	Clipping	1 case of grade II	0	None
V	Clipping	0	1	None
	Endovascular treatment	0	1	None

#### 3.4.2 Type II

**Figure 3:**
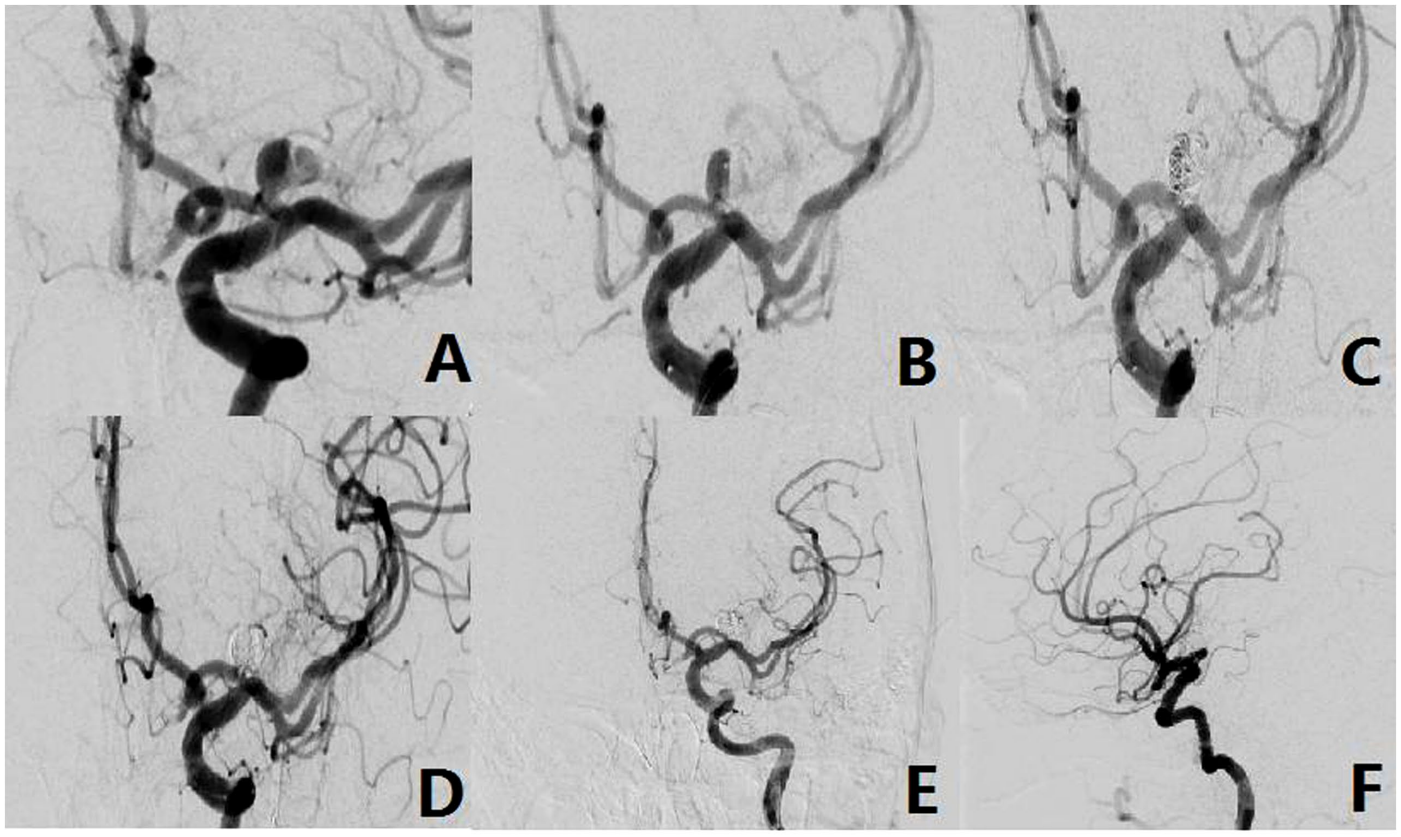
The aneurysm neck is located within the superior wall of the parent vessel (Type-II). **(A)** A 49-year-old female, presented with a non-rupturable aneurysm. DSA revealed that the aneurysm originated from the superior wall of the parent vessel. **(B)** The shape of microcatheter in operation. **(C,D)** Intraoperative situation. **(E,F)** Postoperative radiography situation.

The aneurysm neck was located on the upper wall of the parent artery, and in 6 cases (15.3%), an upward projection of the aneurysm body was observed. All patients within this category underwent endovascular intervention, which included single microcatheter embolization in 2 cases, balloon-assisted embolization in 1 case, stent-assisted embolization in 2 cases, and stent implantation in 1 case involving an unruptured very small aneurysm. Notably, all patients with type II aneurysms were discharged with a favorable prognosis, as depicted in Figure 3.

#### 3.4.3 Type III

**Figure 4:**
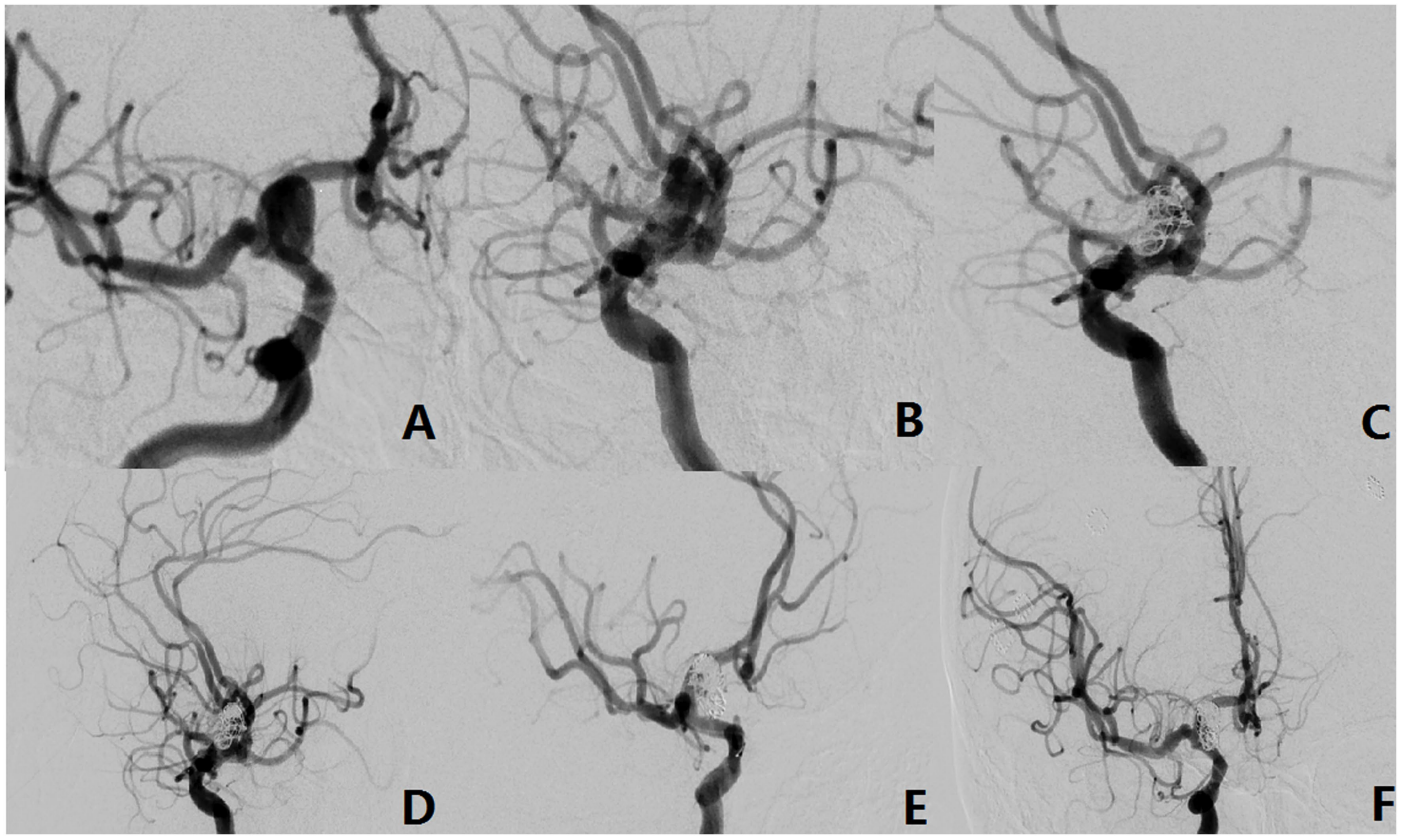
The aneurysm neck is located in the anterior wall of the parent vessel (Type-III). **(A,B)** A 42-year-old male with an unruptured aneurysm. The DSA showed the aneurysm originated from the anterior wall of the parent vessel. **(C–E)** Intraoperative situation. **(F)** Postoperative radiography situation.

The aneurysm neck was located on the anterior wall of the parent artery, and in 3 cases (7.7%), a forward projection of the aneurysm body was evident. Microsurgical clipping was employed in 2 patients, yielding 1 case with a favorable prognosis and 1 case resulting in fatality. Additionally, 1 patient underwent single microcatheter interventional embolization, which demonstrated complete occlusion on immediate postoperative angiography and led to a favorable prognosis upon discharge, as illustrated in Figure 4.

#### 3.4.4 Type IV

**Figure 5:**
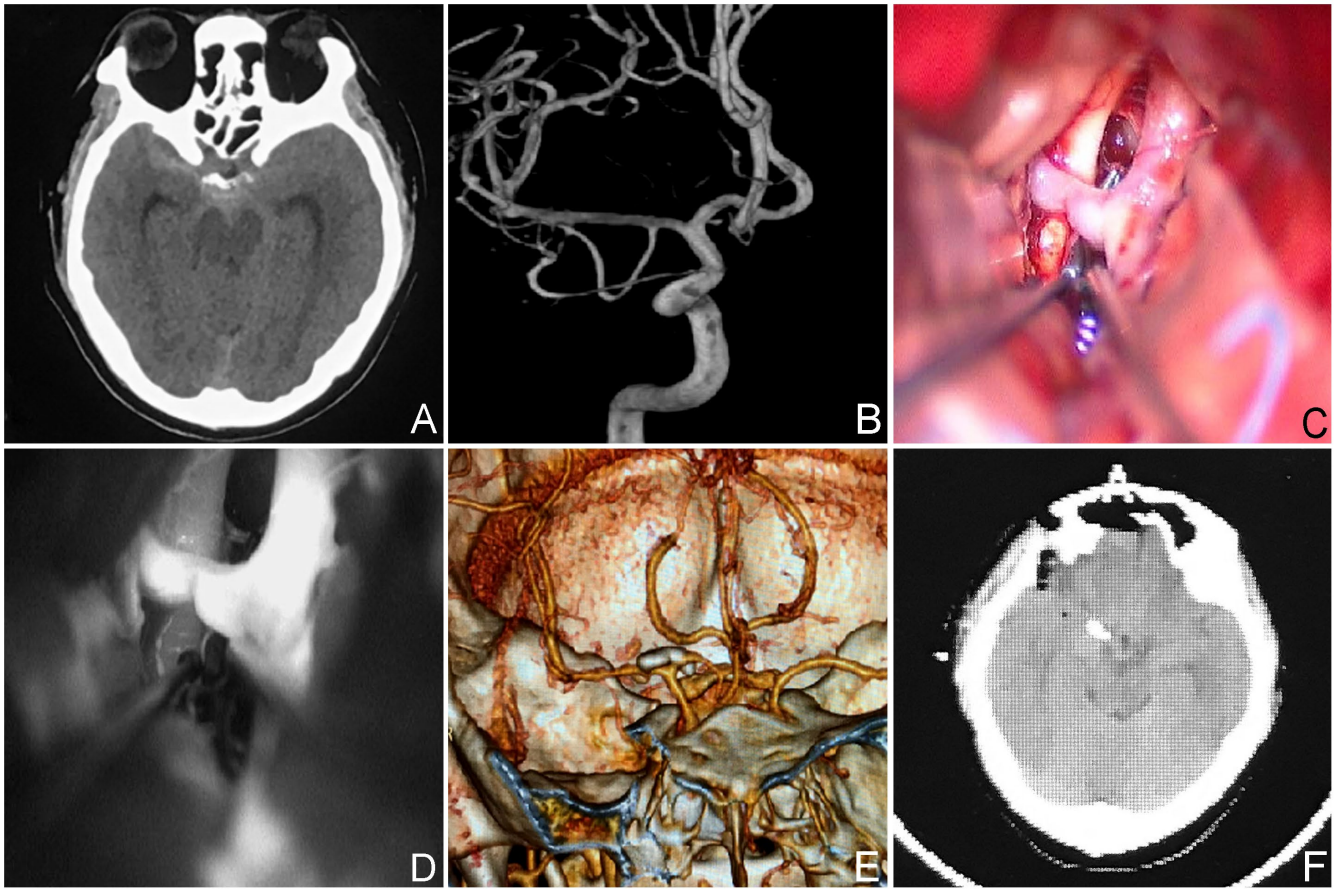
The neck of the aneurysm is situated within the inferior wall of the parent vessel (Type-IV). **(A,B)** A 43-year-old male presented with SAH on a CT scan indicating a ruptured aneurysm. The aneurysm originated from the inferior wall of the parent vessel, as determined by the DSA. **(C,D)** Intraoperative situation. Postoperative CTA **(E,F)** and CT scan.

The aneurysm neck was positioned in the inferior wall of the parent artery, and in 1 case (2.5%), the aneurysm body displayed a downward projection. Microsurgical clipping was conducted, and the patient was discharged with a favorable postoperative recovery, as depicted in Figure 5.

#### 3.4.5 Type V

**Figure 6:**
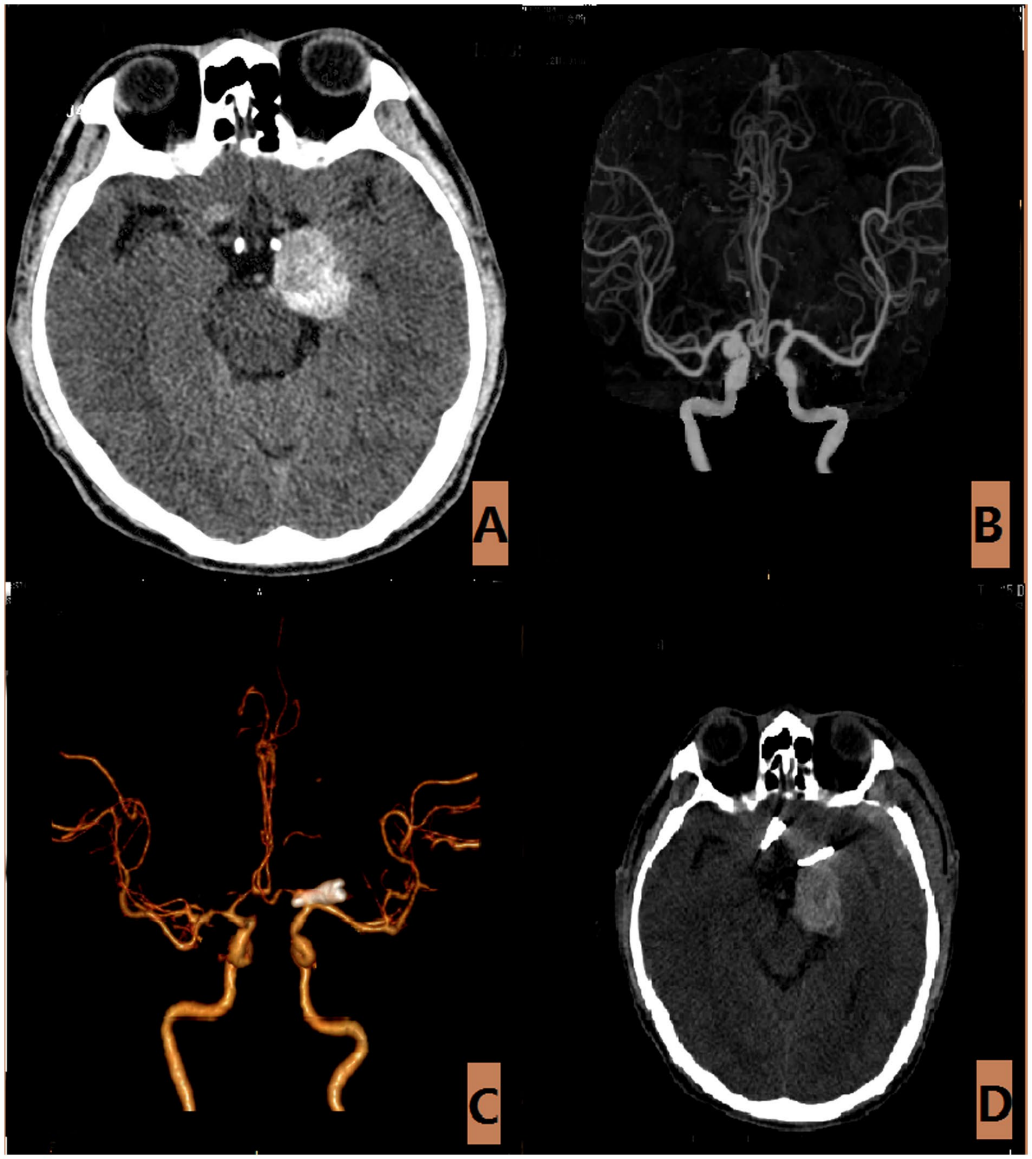
The aneurysm is of the largest variety (Type-V). **(A)** The CT scan of a 60-year-old male with an unruptured large aneurysm, revealed occupying lesions throughout the brain. **(B)** The CTA revealed that the aneurysm measures approximately 2.7 × 6.0 millimeters. **(C,D)** Postoperative CTA and CT scan.

Two cases (5.1%) presented with fusiform or large aneurysms, characterized by neck involvement spanning more than two quadrants of the cross-section of the parent artery and the neck or affected area of the aneurysms is located at the proximal one-third of the A1 segment of the anterior cerebral artery. Microsurgical clipping was performed in 1 case, while embolization assisted by a blood flow diverter device was used in the other case. Notably, all patients in this category experienced favorable recovery and were discharged without any neurological deficits, as illustrated in Figure 6.

For all A1 segment proximal aneurysms in the study group, a comparative analysis of the postoperative 6-month GOS scores between the endovascular treatment group and the microsurgical clamping treatment group showed that the prognosis of the endovascular treatment group was better than that of the microsurgical clamping treatment group (*p* < 0.05) (Table 4). We briefly analyzed the reasons for this result: (1) The microsurgical clamping treatment group had higher postoperative complications than the endovascular treatment group (including brain tissue infarction, frontal lobe hematoma, hydrocephalus, intracranial infection, etc.). (2) Patients in the microsurgical clipping treatment group may have an increased chance of brain tissue damage during surgery due to poor exposure of the aneurysm neck and longer surgical time. (3) During microsurgical clipping of aneurysms, the proximal aneurysm of segment A1 often points backwards and is easily covered by the parent artery, making it difficult to separate the aneurysm neck and surrounding perforating vessels during surgery, which can easily cause secondary damage to the perforating vessels. For all types of patients in the endovascular intervention treatment group, the postoperative GOS score at 6 months was 5 points. In the microsurgical clipping surgery group, 16 patients with a score of 5 (8 cases of IA type; 5 cases of IB type; 1 case of III type; 1 case of IV type; 1 case of V type) and 2 patients with a score of 4 were all IA type in the GOS score 6 months after surgery; two patients with a score of 3 were both type IA; one patient with 1 score is type III.

**Table 4:** Comparison of 6-month GOS scores between all patients in the craniotomy microsurgical clipping group and the endovascular intervention treatment group.

Surgical methods	5	4	3	2	1	*T*-test	*p*-value
Clipping	16	2	2	0	1	−2.118	0.047
Endovascular treatment	18	0	0	0	0		

All patients in the endovascular treatment surgery group showed good occlusion of the aneurysm on angiography after surgery, while most patients in the microsurgical clipping group underwent full brain angiography or cranial CTA during the recovery period, and no obvious recurrence or residual aneurysm was observed. Unfortunately, due to patient compliance and other reasons, a considerable number of patients are still unable to achieve imaging follow-up 6 months after surgery.

## 4 Discussion

Based on literature review, most A1 segment aneurysms are predominantly located at the proximal 1/3 of the segment. This is due to a higher concentration of perforating vessels in this region compared to the distal end (8). Notably, the Heubner recurrent artery, the largest perforator, which frequently courses along the A1 segment and aneurysms originating from the A1 segment are often in close proximity to its origin from the internal carotid artery and the branching point of the anterior choroidal artery (AChA) (9, 10). Consequently, accurate identification and protection of these structures during surgical interventions are crucial to minimize postoperative complications.

Type I classification involves cases where the aneurysm neck is on the posterior wall of the parent artery, with a posterior projection. Further subclassification into Type Ia (posterior inferior) or Type Ib (posterior superior) is made based on the direction of the projection. For both subtypes, microsurgical clipping and endovascular intervention treatments yield comparable prognoses. Thus, either approach can be considered acceptable for patients with unruptured aneurysms or ruptured aneurysms accompanied by Hunt-Hess scores I–II. However, for ruptured aneurysms with higher Hunt-Hess scores (III–V), microsurgical clipping appears more effective, especially in the presence of intracranial hematoma. The use of an expanded peritoneal approach not only facilitates successful aneurysm clipping but also allows for intracranial hematoma removal, reducing intracranial pressure and enhancing patient prognosis.

Presently, primary techniques in endovascular treatment include simple coil embolization, stent implantation, stent-assisted coil embolization, balloon-assisted coil embolization, parent artery occlusion, and contralateral retrograde embolization via the anterior communicating artery. In recent years, the Woven EndoBridge Device WEB (MicroVention) has been increasingly used for endovascular treatment of wide necked aneurysms (11). Some scholars have also applied WEB devices to the treatment of anterior communicating artery aneurysms and explored their impact on the variation of bilateral A1 anatomical structures (12). Unfortunately, we have not found any relevant reports on WEB devices for A1 segment aneurysms. Endovascular treatment for A1 segment aneurysms offers advantages such as minimal damage to perforator vessels and reduced trauma, leading to faster recovery. However, challenges include the selection of working angles due to the spatial configuration of the aneurysm and blood vessel interaction, as well as the difficulty in microcatheter super selection, particularly for aneurysms on the posterior wall. Stabilizing the microcatheter within the aneurysm cavity poses a challenge (13), increasing the risk of residual aneurysms and recurrence. A study by Cho et al. (14) involving 34 patients with proximal A1 aneurysms reported stable occlusion in 91.2% of cases, with varying degrees of residual aneurysms in 8.8% of cases. Furthermore, the average diameter of A1 segment aneurysms was 4.3 mm, primarily consisting of small aneurysms. Managing small aneurysms presents challenges due to their high risk of intraoperative hemorrhage (15). Small aneurysms are difficult to treat and have a high probability of intraoperative bleeding (16). For very small aneurysms, adopting a semi-released state during stent-assisted embolization is recommended to minimize the risk of aneurysm rupture and facilitate coil access (17). Meanwhile, stent assisted embolization has been widely used due to its low recurrence rate, high occlusion rate, and better occlusion progression (18). However, with the development of interventional materials and vascular intervention surgical techniques, blood flow guidance devices are increasingly being used for endovascular intervention treatment of intracranial aneurysms. For large or huge aneurysms, the application of blood flow guided devices (such as Pipeline) will significantly reduce the postoperative recurrence rate compared to traditional balloon assisted embolization or stent assisted embolization. For cases where the diameter of the parent artery is small, such as A1 and A2 segments of the anterior cerebral artery or M1 and M2 segments of the middle cerebral artery, low profile flow diverting stents (FDS), such as the Silk Vista Baby (SVB), the p48MW and small PEDs, are technically feasible for the treatment of intracranial aneurysms, and the prognosis and follow-up results are also satisfactory (19–22).

Microsurgical clipping for type I aneurysms offers advantages such as precise treatment and a high rate of complete occlusion. However, certain drawbacks are associated with this technique. Firstly, the location of type I aneurysms, particularly type Ia, in the posterior wall of the parent artery poses challenges in exposing the neck of the medial lenticulostriate artery, which predominantly arises from this region. Careful separation and protection of this artery are crucial during the operation to prevent damage and nerve function defects post-surgery. Secondly, the proximity of these aneurysms to the end of the internal carotid artery and the origin of the AChA from the internal carotid artery raises the risk of “AChA syndrome” after injury. This syndrome is characterized by contralateral limb hemiplegia, hemisensory disturbance, syntropic hemianopsia, and other symptoms due to involvement of the AChA. Furthermore, infarction in the dominant hemisphere AChA may give rise to “thalamic aphasia,” a condition distinguished by manifestations such as silence, diminished speech pace, and verbal dysfluency (23). Selecting a mini clip suitable for small aneurysm necks is advisable, but in cases involving large aneurysms, brain tissue release after clipping completion may result in twisting of the aneurysm clip, leading to occlusion of surrounding perforating vessels. Concentrating on type I microsurgical clipping within our study cohort, 4 cases manifested scattered patchy cerebral infarctions localized within the ipsilateral frontal lobes and basal ganglia. Among them, 1 case experienced unilateral limb muscle weakness but enhanced with rehabilitation training during the 3-month follow-up, while the remaining 3 cases revealed imaging evidence of cerebral infarction without neurological deficits.

Type II aneurysms, with the aneurysm neck located in the upper wall of the A1 segment projecting superiorly, were treated with endovascular intervention in all cases, resulting in a favorable prognosis with stable occlusion. Challenges included poor stability in microcatheter positioning after super selective access to the aneurysm cavity, leading to the “kicking” phenomenon at the distal tip of the microcatheter during coil filling in two patients, necessitating balloon and stent assistance. One patient underwent simple stent implantation due to difficulties in super selecting the aneurysm cavity, achieving complete occlusion during follow-up. Further investigation is necessary to determine the prognosis of simple stent implantation for very small aneurysms.

For type II aneurysms, it is recommended to shape the microcatheter tip into an “S shape,” “90°,” or “45°” based on the projection direction and vessel morphology during surgery. This facilitates super selection of aneurysms. Of course, microsurgical clipping of the aneurysm is also one of the options. We believe that during the process of separating the aneurysm, the aneurysm body may largely block the aneurysm neck, which brings certain difficulties and risks to the surgery. Therefore, endovascular treatment is our first choice. Because the incidence rate of this type of aneurysm is relatively low, unfortunately, we have no experience of microsurgical clipping at present.

Type III aneurysms have the aneurysm neck situated in the anterior wall of the A1 segment, with the aneurysm body projecting forward. This is a relatively rare occurrence. In this study, microsurgical clipping was performed in 2 cases and endovascular treatment in 1 case. In 1 patient with microsurgical clipping, there was a preoperative Hunt-Hess score of Grade III, and postoperative complications included left frontal lobe hematoma, pulmonary embolism, and cardiac insufficiency, ultimately leading to mortality during follow-up. Clipping type III aneurysms is relatively straightforward due to fewer perforators in the anterior wall; however, the occurrence of postoperative frontal lobe hematoma emphasizes the need for delicate surgical procedures and attentiveness to protecting draining veins and nerve tissue.

Type IV aneurysms have the neck situated in the inferior wall of the parent vessel, with the aneurysm body protruding towards the same wall. This clinical presentation is rare, with only 1 case identified in this group, but the prognosis is favorable, and the patient was discharged. Microsurgical clipping of type IV aneurysms poses challenges due to obscuration by the parent artery at the neck site, limited visual field during dissection and clipping procedures, and potential risk for injury to peripheral perforator vessels. The recommended approach involves initially separating adherent perforators on both sides of the aneurysm before using a clip with a curvature close to 90° that extends along both sides of the aneurysm blade.

Ensuring attachment of the curved portion of the clip to the inferior wall of the anterior cerebral artery and placement of the blade tip at its anterior edge is crucial for effective treatment of type IV aneurysms. Confirmation of complete clipping should be obtained after closure to ensure treatment efficacy. Two cases reported by Cho et al. opted for interventional treatment, resulting in successful treatment and subsequent discharge (6). Therefore, endovascular treatment remains a viable option for type IV aneurysms.

Type V aneurysms, characterized by fusiform or large size with neck involvement of the parent artery in more than two quadrants or aneurysm size greater than 25 mm, are relatively rare (24). In this study, 2 cases of unruptured giant aneurysms were identified, with 1 presenting a substantial diameter of approximately 3.0 cm. The patient sought medical attention at the hospital due to persistent dizziness and progressive vision loss over a six-month period. Head CT revealed intracranial space-occupying lesions, and CTA indicated that the initial diameter of the A1 segment aneurysm was around 6.0 mm. During the operation, it was confirmed that there was partial thrombosis in the tumor cavity and hyperplasia and calcification in the tumor wall.

Endovascular treatment, particularly with the advent of flow diverter devices, represents a viable option for giant aneurysms. In this study, another giant aneurysm underwent successful flow diverter-assisted embolization and exhibited favorable postoperative recovery outcomes. However, it is noteworthy that this method has limited decompression efficacy. Therefore, endovascular interventional treatment may be considered for giant aneurysms without complications such as optic nerve compression or ischemic cerebrovascular disease resulting from space-occupying effects.

Comparative analysis of prognosis between endovascular treatment and microsurgical clipping treatment for the same type of aneurysm: due to the small proportion of A1 segment initial aneurysm in all intracranial aneurysm cases, it is a relatively rare special case. The reasons for choosing surgical methods are also multifactorial, including the surgeon’s habits and personal experience, the willingness of the patient’s family, and the financial situation. So unfortunately, we have not obtained comparative results on the prognosis of all A1 segment initial aneurysms of the same type using different surgical methods. There were a total of 6 cases of type II aneurysms, all of which received endovascular intervention treatment. There were no cases of microsurgical clipping treatment, and all had a good prognosis. A total of 1 case of type IV aneurysm was treated with microsurgical clipping, and there were no cases of endovascular intervention treatment, all with good prognosis. We conducted statistical analysis on the prognosis of endovascular intervention and microsurgical clipping treatment groups for type Ia and type III aneurysms, respectively. The statistical analysis results showed no significant statistical difference between the two groups (Table 2) (results calculated using Fisher’s exact probability method). However, we speculate that this may be due to limitations in sample size. There were a total of 18 cases of type Ia aneurysms, including 12 cases of microsurgical clipping, 6 cases of endovascular treatment, and 2 cases of poor prognosis among the 18 cases, all from the microsurgical clipping group. One case was secondary hydrocephalus after ventricular hemorrhage, and one case was secondary intracranial infection caused by hydrocephalus, both of which were related to open surgery. A total of 4 cases of surgical related cerebral infarction occurred during microsurgical clipping surgery, of which 3 cases were asymptomatic cerebral infarction. However, only one asymptomatic cerebral infarction occurred in the endovascular treatment group (Table 3). There were a total of 3 cases of type III aneurysms, including 2 cases of microsurgical clipping and 1 case of endovascular treatment. Among the 3 cases, 1 had a poor prognosis and came from the microsurgical clipping group. The poor prognosis was due to postoperative frontal lobe hematoma. Although further surgery was performed to remove the hematoma and reduce pressure, the patient still had a poor prognosis, which was considered to be related to traction on the frontal lobe and obstruction of venous return during surgery (Table 3). There were a total of 9 cases of type Ib aneurysms, including 5 cases of microsurgical clipping and 4 cases of endovascular treatment, all with good prognosis. There were a total of 2 cases of V-type aneurysms, including 1 case of microsurgical clipping and 1 case of endovascular treatment, with good prognosis.

## 5 Limitations

The limitations of this study include the lack of angiographic follow-up for a number of patients, which may have affected our ability to fully assess the long-term outcomes of the treatments. Additionally, the small group sizes for the anatomical classification limited our capacity for statistical comparisons between surgical and interventional patients within each group. The reliance on self-reported outcomes may introduce subjectivity and potential bias. Furthermore, the need for larger studies is highlighted to confirm our findings and to explore the efficacy of different treatment modalities more comprehensively.

## 6 Conclusion

Aneurysms originating from the proximal A1 segment of the anterior cerebral artery are relatively uncommon intracranial aneurysms, distinguished by unique features. We believe through literature review and case analysis that endovascular intervention may have certain advantages in reducing postoperative complications and prognosis compared to microsurgical clipping surgery for the treatment of proximal segment A1 aneurysms of the anterior cerebral artery. The postoperative GOS scores of the two have significant statistical significance. The anatomical classification of aneurysms in this region may be of help to develop effective treatment strategies. Further studies with larger number of cases are needed to confirm these results. We hope that our summary and analysis can provide guidance and assistance for neurosurgeons in the diagnosis and treatment process, in order to reduce the occurrence of postoperative complications in patients and improve their prognosis.

## Data availability statement

The raw data supporting the conclusions of this article will be made available by the authors, without undue reservation.

## Ethics statement

This study was conducted in accordance with the declaration of Helsinki and with approval from the Ethics Committee of the Second Hospital of Hebei Medical University. The studies were conducted in accordance with the local legislation and institutional requirements. The participants provided their written informed consent to participate in this study.

## Author contributions

X-mL: Conceptualization, Formal analysis, Writing – original draft, Writing – review & editing. X-lS: Conceptualization, Writing – original draft, Writing – review & editing. KT: Data curation, Writing – review & editing. CZ: Data curation, Writing – review & editing. X-sL: Data curation, Writing – original draft. LZ: Formal analysis, Writing – review & editing. X-lW: Data curation, Formal analysis, Writing – review & editing. H-lD: Data curation, Formal analysis, Writing – review & editing. Y-hH: Formal analysis, Writing – original draft, Writing – review & editing. J-lW: Conceptualization, Writing – original draft, Writing – review & editing.
